# Treatment of Gonorrheal Rheumatism

**Published:** 1899-12

**Authors:** 


### Treatment of Gonorrheal Rheumatism.

Archibald E. Garrod says, in discussing the treatment of this disease, that the first object in its treatment is the cure of the urethral discharge in which it has its origin; with this end in view the ordinary remedies should be applied both by the mouth and in the form of injections.

With regard to the medicinal treatment of the disease little can be said that is at all encouraging. The salicylic drugs and colchicum alike are without appreciable effect upon the articular lesions, and lapse of time is the chief means of cure. Iodid of potassium is the drug which is most relied on, and he is in the habit of giving a mixture containing quinine with an alkali, together with the idodid, and he believes that this line of treatment is by no means without good effect. If the pain be severe, opium may be administered. In severe cases the inflamed joint may need a splint, and th eapplication of an ice bag usually affords relief. When the effusion into the articular cavity is very abundant, paracentesis may be called for; and in the rare instances in which a joint suppurates, further surgical interference will of course be required.

The great tendency of the formation of fibrous adhesions must never be lost sight of; if the joint be put on a splint, care must be taken that if ankylosis should result, the limb may be fixed in as advantageous a position as possible. Although complete recovery of the affected joints is the rule, at least in the milder cases, some degree of stiffness often remains, which calls for passive movement under an anæsthetic; the minor degrees of residual pain and stiffness may often be got rid of by a course at some mineral water resort where external treatment is carried out. It is, of course, only from the external application of mineral waters that any results can be looked for.

The following prescriptions will be found efficient in the treatment of gonorrhœal rheumatism:

℞ Ung. belladonnæ
Ung. hydrargyri, aa..........................℥i

M. Sig.: Apply externally to the affected joint and bandage snugly.

℞ Guaiamar..................................ʒii

Ung. belladonnæ

Ung. hydrargyri, aa.........................ʒiv

M. Sig.: To be rubbed freely into the joint, which is then covered with cotton and bandaged.

℞ Guaiacol
Glycerin, aa..........................℥ss

M. Sig.: Apply to joint, cover with cotton and bandage as directed above.—*Journal A. M. A.*

